# Fine Cellulosic Materials Produced from Chemical Pulp: the Combined Effect of Morphology and Rate of Addition on Paper Properties

**DOI:** 10.3390/nano9030321

**Published:** 2019-03-01

**Authors:** Julie Bossu, René Eckhart, Chiara Czibula, Armin Winter, Armin Zankel, Wolfgang Gindl-Altmutter, Wolfgang Bauer

**Affiliations:** 1 JRU IATE 1208—CIRAD/INRA/Montpellier Supagro/University of Montpellier, 2 Place Pierre Viala, Bat 31, CEDEX 01, F-34060 Montpellier, France; 2 Institute of Paper, Pulp and Fibre Technology, NAWI Graz, Graz University of Technology, Inffeldgasse 23, 8010 Graz, Austria; chiara.czibula@tugraz.at (C.C.); wolfgang.bauer@tugraz.at (W.B.); 3 Department of Materials Sciences and Process Engineering, Institute of Wood Technology and Renewable Materials, University of Natural Resources and Life Sciences Vienna, Konrad-Lorenz-Straße 24, 3430 Tulln, Austria; armin.winter@boku.ac.at (A.W.); wolfgang.gindl@boku.ac.at (W.G.-A.); 4 Institute for Electron Microscopy and Nanoanalysis, NAWI Graz, Graz University of Technology and Centre for Electron Microscopy, Steyrergasse 17, 8010 Graz, Austria; armin.zankel@felmi-zfe.at

**Keywords:** papermaking, microfibrillated cellulose, fines, morphology, paper strength, fiber network

## Abstract

Among bio-based reinforcement additives for paper existing on the market, microfibrillated cellulose (MFC) turned out to be a promising material, showing outstanding potential in composites science. Its relevance in papermaking as a new family of paper components was suggested more recently. There remains a number of constraints limiting the promotion of their use in papermaking, mostly related to their high cost and effect on dewatering resistance. Also, contrasting results reported in the literature suggest that the effect of fibrillation rate and quantity of such cellulosic additives in a furnish on the technological paper properties needs further research. The purpose of this study is to produce and characterize different MFC-like fine fibrous materials of varying particle size and degree of fibrillation from the same batch of pulp through mechanical treatment or fractionation. The effect of the thus obtained fine fibrous materials on paper properties is evaluated with respect to their concentration within a fiber furnish. We compared: (i) a mixture of primary and secondary fines isolated from the pulp by means of a purpose-built laboratory pressure screen; (ii) MFC-like fine fibrous materials of increasingly fibrillar character obtained by refining and subsequent steps of high-pressure homogenization. The morphology of the different materials was first characterized using flow cell based and microscopic techniques. The thus obtained materials were then applied in handsheet forming in blends of different proportions to evaluate their influence on paper properties. The results of these experiments indicate that all these products lead to a substantial decrease in air permeability and to improved mechanical properties already at low concentration, independent of the type and morphological character of the added fine cellulosic material. At higher addition rates, only highly fibrillated materials allowed a further considerable increase in tensile and z-strength. These observations should help to allow a more targeted application of this new generation of materials in papermaking, depending on the desired application.

## 1. Introduction

In a competitive environment resulting from globalization and various other economic factors, balancing sustainability and profitability is a challenge but also a huge opportunity for the paper industry. To address this issue, papermaking manufacturers continually intensify their efforts to reduce production costs while attempting to improve the quality and environmental performance of their end products. A possible way to produce new competitive products is to develop efficient strengthening additives to meet the requested mechanical properties of the final product at a reduced raw material input.

Precisely in this context, cellulosic nanomaterials, such as cellulose nanofibers and nanocrystals, as well as the coarser microfibrillated celluloses (MFC) have emerged over the last twenty years as promising and sustainable reinforcement materials, showing outstanding potential in material sciences. While their possible use in composite materials based on polymeric matrices has led to intensive research [1,2,3], their application in papermaking, however, has received less attention. Recent studies show interesting results in the improvement of paper properties using MFCs as additives [4,5,6,7,8]. Thanks to their intrinsic high mechanical strength along with good flexibility and high potential to interact with cellulosic fibers, authors have reported a significant improvement of paper properties by the application of highly fibrillated additives. It is has also been reported that pulp origin and treatment, as well as both type and amount of added MFC, affect the final paper overall properties, leading to sometimes contrasting results [5,6,7]. However, this wide range of variability also represents a good opportunity to tailor the performances of paper if the effects of MFC additives become better understood.

While such arguments motivate the interest of considering MFC as a new family of paper components, their scale of production is still limited and below expectations. One major obstacle is the high buying-in price of MFCs, originating from the high energy consumption involved during their production [9,10]. To take advantage of the reinforcement potential of such materials in papermaking, development of new solutions to produce highly fibrillated additives directly on site is needed to maintain a satisfying balance between costs and benefits. As an alternative to commercial MFC, fine fibrillar materials generated in the pulp and paper production processes—if separated from the pulp and applied in a targeted way—could be a promising resource to achieve similar results as with MFC addition and improve the added value of paper products [11,12].

By using fractionation and different mechanical processing steps, different fine cellulosic materials can be produced, covering a wide range of particle sizes and degrees of fibrillation. Fine fibers fragments produced along the pulping and papermaking process can be separated from the pulp through fractionation [13] and are reported to have a strong impact on strength properties of paper [14,15,16]. Another well-established approach is to mechanically process virgin pulp fibers to increase their fibrillar character through high-pressure homogenization. Since the introduction of MFC by Turbak [17], this technique stands as a simple and relatively low-cost processing solution to effectively produce MFC [6,18,19].

The objectives of this work are to (i) produce MFC-like additives from the same batch of pulp, through filtration and mechanical treatments, resulting in a broad distribution of morphological properties (diameter, length and fibril area) and (ii) realize blends of varying concentrations mixing the produced materials to a reference pulp and produce handsheet samples to test the effect of additives on paper technological properties. The results obtained are expected to provide a better understanding of the mechanisms behind the reinforcing effect of fine cellulosic additives on the paper network, depending on their nature and quantity.

## 2. Material and Methods

### 2.1. Preparation and Characterization of Fibrillated Additives

The same batch of softwood bleached Kraft pulp (ORION, heinzel pulp—Zellstoff Pöls AG, Pöls, Austria) was used to produce all the materials used in this study.

A sample of this pulp was refined in an industrial double-disc refiner to 38SR and then fractionated using a lab-scale pressure screen equipped with a perforated plate (hole diameter 100 µm) to separate the primary fines already contained in the unrefined pulp and the secondary fines produced during refining from the fibers [20]. As the major part of the obtained fines belongs to the class of secondary fines, this term is used further for this material. The pulp was recirculated in the screening system until the remaining volumetric fines content in the pulp was below 0.5% (measured with L&W Fiber Tester+). The fines were allowed to settle for three days, before the supernatant was removed and a concentration of approximately 1% w/w was reached. 

The unrefined pulp from the same production batch was used to produce materials of increasingly fibrillar character. For this purpose, the unrefined pulp was mechanically processed by classical refining using a Valley Beater (Valley Laboratory Beater PTI Type, 230 V–50/60 Hz–0.8 kV, PTI, Laakirchen, Austria) and subsequent application of different routines of homogenization with a high-pressure homogenizer (APV 1000, 1000 Bars–30 Hz, APV, Tczew, Poland). In case of these refined and homogenized materials, no fractionation was applied. The different materials produced in this study and used as additives to the pulp are summarized in Table 1.

### 2.2. Characterization of Fine Cellulosic Materials

The morphology of the fibers and fibrils from the samples was determined using a L&W Fiber Tester+. Frequency distribution, average fiber length and ECD (Equivalent Circular Diameter) were calculated (weight weighted) using a MATLAB routine processing the raw data obtained by the device. The fines fraction of the different cellulosic materials produced in high-pressure homogenization was measured using a Britt Dynamic Drainage Jar according to TAPPI Test Method T-261 cm-10 (fines fraction by weight of paper stock by wet screening).

Highly fibrillated material is difficult to detect using flow imaging. The contrast of such highly swollen particles in the flow cell images from the L&W Fiber Tester+ is sometimes too low, which strongly affects the detection of the highly fibrillated parts. Thus, in order to accurately identify the fibrillar content of the cellulosic fine materials, additional microscopic samples were produced, using a specific staining procedure to improve the identification of the fibrillar content. The different cellulosic materials were diluted to 0.01% w/w and dyed using a combination of a tall oil–water emulsion (0.2 wt.% in water) with a methylene blue solution (1 wt.% in water), reaching a uniform coloration [21]. Microscope slides were prepared from the stained samples and characterized using a method described in Mayr [21], using a conventional transmission light microscope (Leica 301-371.010) equipped with a standard CCD camera (Jai AM-200GE/AB-200GE) and an automated stage control (Märzhäuser Multicontrol 2000). Image analysis was performed using the open source software ImageJ (Bethesda, Maryland, USA) for automated capturing of 700–800 single images per microscope slide (1600 × 1200 pixels per image; image area: 1380 × 1035 μm^2^). From these images, the Total Detectable Area and the Flake-like Area has been identified using thresholding. The Fibril Area (i.e., the microfibrillar content of the analyzed fraction) was then calculated as follows:

$$\mathrm{Fibril}\mathrm{Area}\left[\%\right]=\frac{\mathrm{Total}\mathrm{Detectable}\mathrm{Area}-\mathrm{Flake}-\mathrm{like}\mathrm{Area}}{\mathrm{Total}\mathrm{Detectable}\mathrm{Area}}.$$

**Figure 1:**
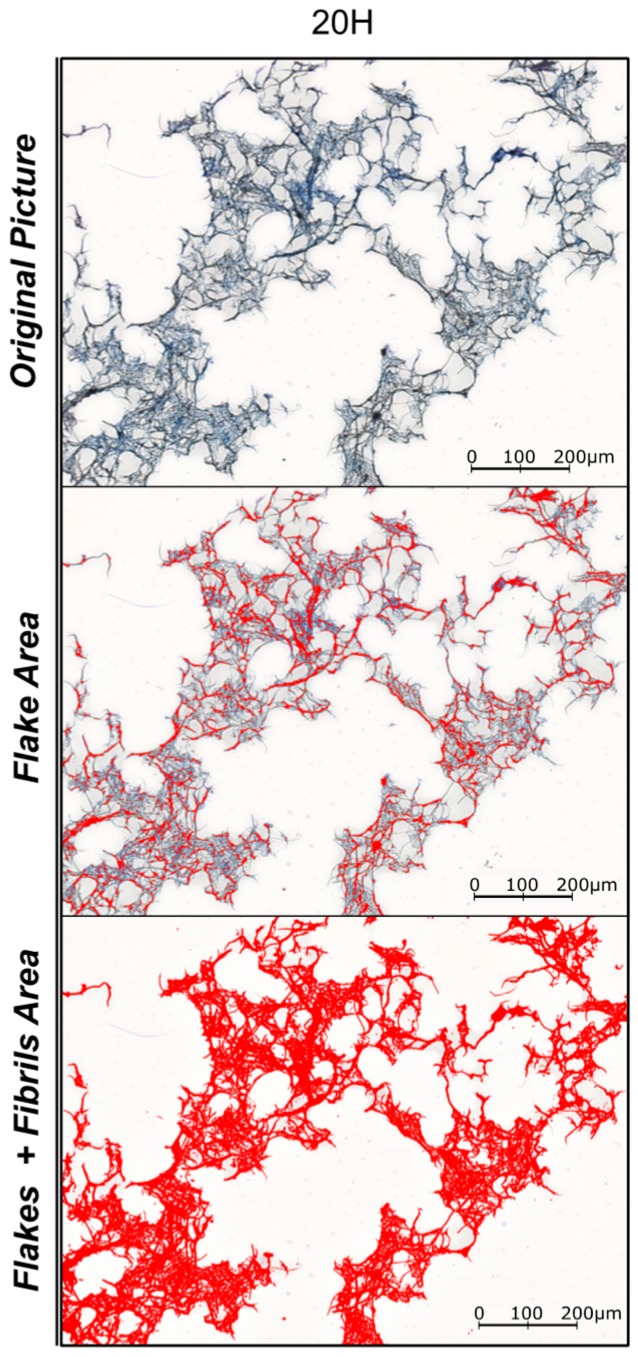
Example of the image analysis processing using ImageJ Software to identify Flakes and Total Detectable Area in one of the 800 pictures realized per samples, sample 20H.

An application of this method is illustrated on Figure 1, where the discrimination between Fibril and Flake-like Area is presented for the sample 20H. Finally, the fines fraction of the different products was measured using a Britt Jar (T-261 cm-10).

### 2.3. Handsheet Preparation and Paper Testing

The same softwood bleached kraft pulp used for production of the materials listed in Table 1 was also used in the blends as the main component of the furnish. For this purpose, the pulp was refined using a PFI - mill to reach mechanical properties relevant in paper production (ISO 5264-2; 2000 revolutions; SR°: 16). This refined pulp also represents the reference for the comparison to the blends with the fine fibrous materials. The produced materials were added to the PFI-refined pulp in different proportions of 1%, 2%, 4%, 7% and 10% (Except for VB where blends where only prepared at 4%, 7% and 10% of addition due to lower amount of available material). The thus produced furnish blends were then mixed using a disintegrator (DIN EN ISO 5263-1) for 25 min. 

Handsheets with a basis weight of 60 g/m^2^ were prepared on a Rapid–Köthen sheet former (ISO 5269-2:2004) using white water recirculation [22] in order to guarantee the desired cellulosic fines content in the sheets. The first five sheets were discarded until a stable fines content in the sheets was achieved. Seven sheets were formed and wet pressed (150 bar, 90 s) between two blotting papers, directly after sheet formation, to limit the influence of capillary forces and their effects on sheet consolidation and to approach industrial production conditions.

The resulting handsheets were conditioned for 24 h in a climate room (23 °C; 50%RH) before testing. Bendtsen air permeability (ISO 5636-3:2013), tensile index (EN ISO 1924-2) and z-strength (Scott Bond, ISO 15754:2009) were measured on the handsheets. Data analysis was performed using R program (R Development Core Team, 2008). The surface topography of the different papers was investigated using low-voltage scanning electron microscopy (LVSEM, Everhart-Thornley detector for the detection of secondary electrons; Zeiss Sigma 300, Oberkochen, Germany) using the method described in Fisher et al., 2014. The samples were cut (1 cm × 1 cm), then attached to SEM stubs using a double-sided conductive carbon tape, and imaging (magnification 500×) was performed at an acceleration voltage of 0.65 kV.

## 3. Results and Discussion

### 3.1. Morphological Properties - Flow Cell-based Device (L&W Fibre Tester+)

**Figure 2:**
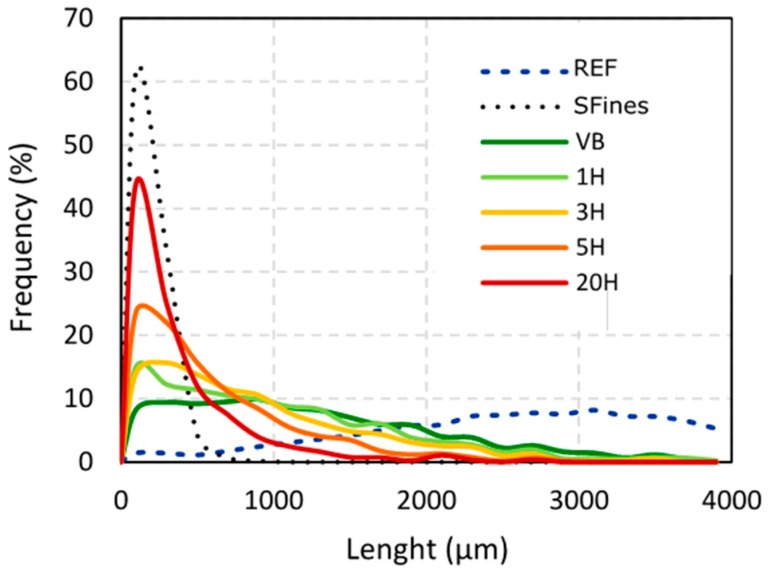
Average distribution of length of the different materials obtained using L&W Fiber Tester+.

Figure 2 presents the distribution of length (weight weighted) of all the materials from Table 1 detected using a L&W Fiber Tester+ in comparison to the reference refined pulp.

The reference pulp fibers show a broad length distribution (Figure 2). The maximal frequencies recorded do not exceed 9%, evidencing an important variability, but most part of the fibers are characterized by a high length (2478.3 µm in average).

**Figure 3:**
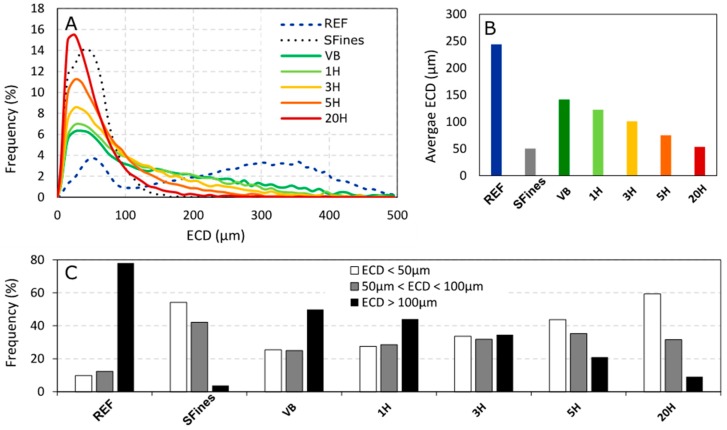
(**A**) Distribution of Equivalent Circle Diameter (ECD) for the different materials, obtained using L&W Fiber Tester+; (**B**) Comparison of the average values of ECD; (**C**) Detail of the partials frequencies of fibers detected per class of ECD.

For ECD, two peaks can be identified for the refined pulp, with a minor amount of smaller fibers (frequency peak of 3.7% for an average ECD of 55 µm) and a larger amount of larger fibers (frequency peak of 3.4% for an average ECD of 355 µm) (Figure 3A). It is likely that the smallest fractions observed here originate from pulp processing, being the secondary fines present in the refined pulp. This assumption is supported by the fact that the average ECD of these smaller fibers is close to the one observed for SFines.

After two hours of beating in the valley beater (VB), the length distribution, as expected, changes considerably compared to the refined pulp, with the largest amount of fibers now being much shorter (Figure 2). Indeed, the average length of VB fibers is already only the half (1226.9 µm in average) compared to the unrefined pulp. Taipale [23] also observed a decrease in fiber length with increasing beating time but less marked. Still, in our case, while the length of the fibers is substantially reduced, there are still long fibers remaining in the material with 14% of the detected fibers being longer than 2000 µm. The average ECD of the VB samples was measured to 141.5 µm, which is the double of the result reported by Retulainen [11].

For the cellulosic materials resulting from valley beater refining and subsequent high-pressure homogenization treatment, an increasing number of homogenization passes substantially increases the amount of short fibers/fines (from 1H to 20H, the proportion of fibers under 100 µm increases from 15.1 to 44.1%) (Figure 2). The average fiber lengths calculated from the distributions of the different materials are presented in Table 2. 

Regarding the ECD distributions, the same conclusions can be drawn (Figure 3A). Also, a gradual decrease of average ECD can be observed from VB to 20H (Figure 3B), highlighting a clear linear correlation between the intensity of the mechanical treatment and the decrease of the fractions size.

The highly treated 20H shows an average ECD value of 53.6 µm. Fischer [12] reported similar values for industrial MFC grades (52.11 µm), which tends to confirm that the additives produced in this study are comparable with cellulosic microfibrils.

Finally, the secondary fines (SFines) present the shortest fibers (185.3 µm in average) with the narrowest distribution. Since this material results from fractionation through a pressure screen, it is natural to observe the narrowest distribution, since long fibers have been separated. SFines also show the lowest values for average ECD (50.6 µm in average), being even lower than the one observed for 20H (53.6 µm in average). The average ECD for SFines we obtained here is a little bit higher than the one reported by Retulainen [11] (40 µm), but in accordance with more recent work from Mayr [20] for similar pulp (47 µm). As the average values we measure with L&W Fibre Tester+ result from a distribution, it is interesting to discriminate the fibers per class of ECD (Figure 3C). Here, one can observe that SFines, as expected, have the lowest amount of large fibers (with ECD > 100 µm) because of the fractionation step, while 20H still shows 9% of fibers with an ECD > 100 µm. Yet, 20H shows a larger amount of fibers belonging to the group of the smallest fibers, with 59% of fibers having an ECD < 50 µm, which is also observable on the ECD distribution graph (Figure 3A). The intensive mechanical treatment obviously produced a much smaller material compared to the fines separated after industrial refining.

### 3.2. Fines Fraction and Morphological Properties - Microscopic Method

**Figure 4:**
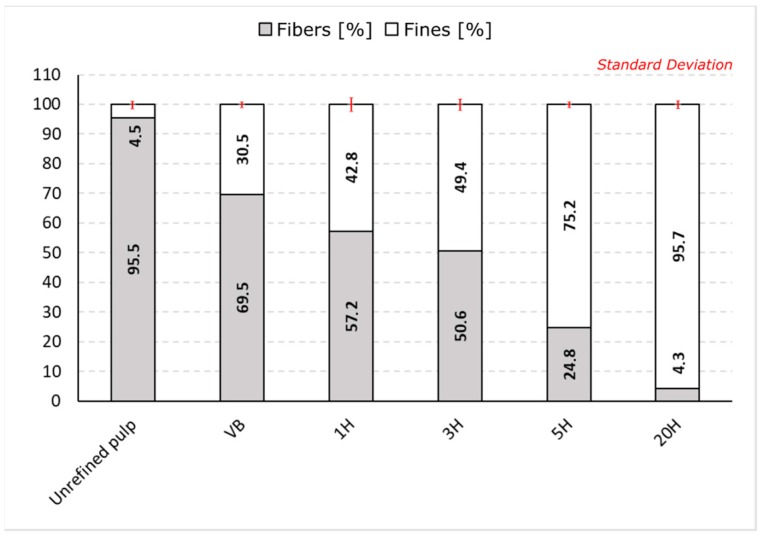
Fibers (white area) and Fines (grey area) fractions measured for the high-pressure homogenized samples (3 samples tested), using Britt Jar system.

For the fine fibrillated materials produced with high-pressure homogenization, the fines fraction has been evaluated using Britt Jar according to the TAPPI Test Method T-261 cm-10. In this method, the separation of the fine particles is performed through wet screening and the fines fraction is defined as the particles that pass a round hole 76 μm in diameter (i.e., a nominally 200 mesh screen). The untreated pulp shows a fines content of 4.5% (Figure 4). After refining the pulp in the Valley Beater, the fine content is more than 6 times higher than in the control sample, with 30.5%. 1 to 3 steps of homogenizations led to an additional increase in the fines content value, with 42.8% and 49.4% of fines content for 1H and 3H samples respectively. In the case of the samples 5H and 20H, resulting from the most intensive mechanical treatment, fines are the main components, representing 75.2% and 95.7% of the material.

**Figure 5:**
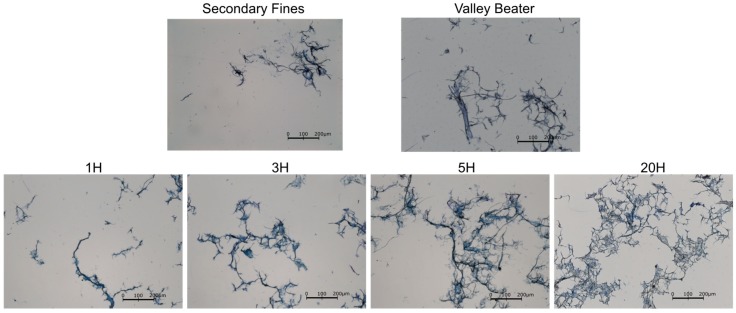
Microscopic Images of the different stained fibrous materials using a combination of tall oil–water emulsion with methylene blue solution and observed with a conventional transmission light microscope (Leica 301-371.010).

Figure 5 provides a qualitative comparison of the microscopic images obtained from all the additives used in this study. Here, it can be observed that even fine fibrils are identifiable and well contrasted, validating the method used for staining. 

Because of fractionation, only small particles are visible for secondary fines, which rarely exceed 100 µm in length. This observation matches the results obtained with L&W Fiber Tester+ where the fractions under 100 µm of length represented 61.8% of the detected particles. Longer particles are observable in the images corresponding to the fine cellulosic materials obtained from mechanical treatment. These images illustrate both the expected gradual reduction of fiber length and the increase of fine elements when increasing the intensity of the mechanical treatment. In the image for VB, a fraction of an untreated fiber section is mixed with fine material. In the image of 20H only fines elements are observable, the proportion of small particles being the dominant in this sample (95.7% of fines accordingly to Britt Jar measurements).

**Figure 6:**
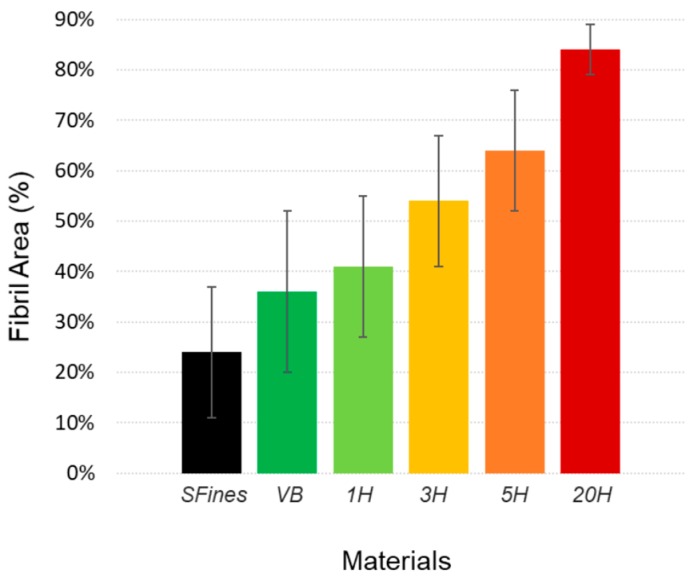
Result of the average Fibril Area for each additive resulting from the image analysis of the stained microscopic samples (average from 700 to 800 images analyzed per sample).

The result of the analysis of the microscopic samples using automated image analysis (average of the results obtained for 700 to 800 images analyzed per sample) highlights a linear increase of fibril area from secondary fines to 20H, confirming the previous qualitative observations on microscopic images (Figure 6). Compared to the fines fraction measured with a Britt Jar, this method enables to discriminate more precisely between fibrillar and flake like material, which allows to quantify the highly fibrillated part of the material expected to bring more adhesion with the fibers when mixed to the refined pulp. 

### 3.3. Effect of the Fine Fibrillar Material on Paper Thickness and Air Permeability

By the addition of the fine fibrous materials to the furnish the increase in specific surface area is assumed to promote the formation of fiber–fiber bonds consolidating the paper structure and increasing density. Indeed, at constant basis weight, one can observe a reduction in paper’s thickness (data not shown).

**Figure 7:**
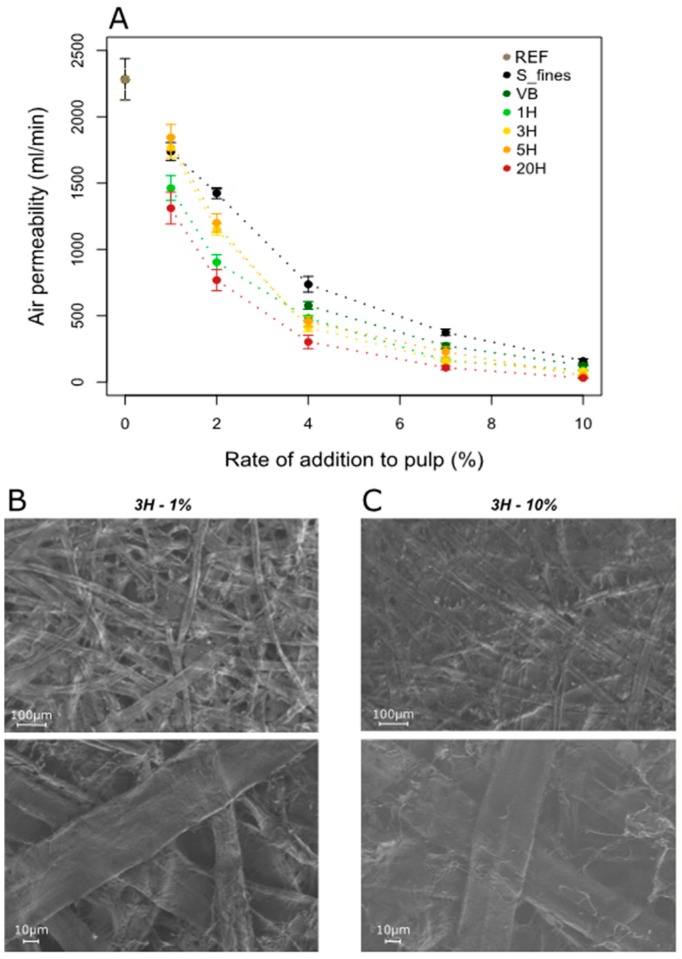
(**A**) Influence of the nature and quantity of the different additives on paper air permeability measurements; (**B**,**C**) SEM images of the topography of composite handsheets realized with 1% and 10% of 3H (magnification 100× on the top image and 500× on the bottom image, with an acceleration voltage of 0.65 kV).

This phenomenon results in a significant decrease in air permeability of paper, a result which has already been reported by other authors [23,24]. In our experiments, the reference paper produced with reference pulp without the addition of fine material shows an air permeability of 2283 mL/min (Figure 7A). Upon addition of the added materials, all mixtures showed a substantial decrease compared to this initial value. At 1% of addition to the pulp, all the different materials already induced a decrease of at least −19.2% from the initial value. One can also observe that the effect of adding fibrillated material to the pulp already causes a significant reduction of air permeability at a low rate of addition with the air permeability being reduced by −78.2% on average already at 4% addition rate, regardless of the nature of the added material. Sehaqui [25] also showed that the initial increase of TI up to 2% of MFC addition to the pulp corresponds to the largest decrease of paper porosity and largest increase of density.

At a higher addition rate, air permeability decreases further but the effect is not as pronounced. Finally, for 10% of addition, the value reaches ~90ml/min, i.e., a total decrease of −96% on average.

These effects of densification of the paper structure are also visible in the SEM images where a clear difference in the paper structure is observed when increasing the quantity of additives (Figure 7B,C). Here, for the additive 3H, the increase of the addition rate from 1% to 10% leads to a filling of the voids between the fibers and leads to a denser structure and smoother surface, resulting in a significantly lower air permeability.

Regarding the specific effect of each material, the most fibrillated material 20H has the most significant effect on air permeability. Comparing all additives shows that the higher the fibril area, the better the air barrier properties. This observation highlights the fact that increasing the fibrillation rate of such additives leads to a better closing of the paper structure. This may be related to the relatively large and highly fibrillated structures shown in the microscope images for highly fibrillated material (Figure 5). The size distribution as it is measured using the L&W Fibre Tester does not correlate well with air permeability. One would suspect the SFines—showing the smallest particles—would be most efficient in closing the sheet. On the contrary VB, which is characterized by a larger amount of longer and thicker fibers compared to SFines, leads to a lower air permeability compared to SFines, apparently because of its higher fibril area. Increasing fibrillation is suspected here to decrease air permeability by increasing pore tortuosity and decreasing pore area and connectivity. Indeed, fluid and gas transportation in the fiber network closely relates to the porosity, the ratio of void volume and total volume of paper [26]. Su [27] also suggest the occurrence of such mechanism with MFC in composites structures: the authors reported that the air permeability for MFC composites is much lower than those from refined fibers, even at comparable densities and that this trend is more obvious at high MFC content, complying with the less porous surface morphology observed by microscopy.

Here, it is interesting to note that the compact structure of composites papers made with highly fibrillated additives indicate distinctive barrier behavior from that of porous papers, which can be of interest for food packaging applications for example. The large range of air permeability reported here highlights the potential of using fine cellulosic additives to tailor paper barrier properties depending on the requirements of the final application like paper pouches, bags and boxes. Here, further analysis of factors like the oxygen transmition rate as well as water and grease permeabilities should also be tested to validate the interest of these materials for biopackaging applications. On the other hand, it has also to be considered that the densification of paper can decrease paper bending stiffness, which is an important property in many paper grades, as pointed out by Sehaqui [25].

### 3.4. Effect of the Additives on Paper Mechanical Properties

**Figure 8:**
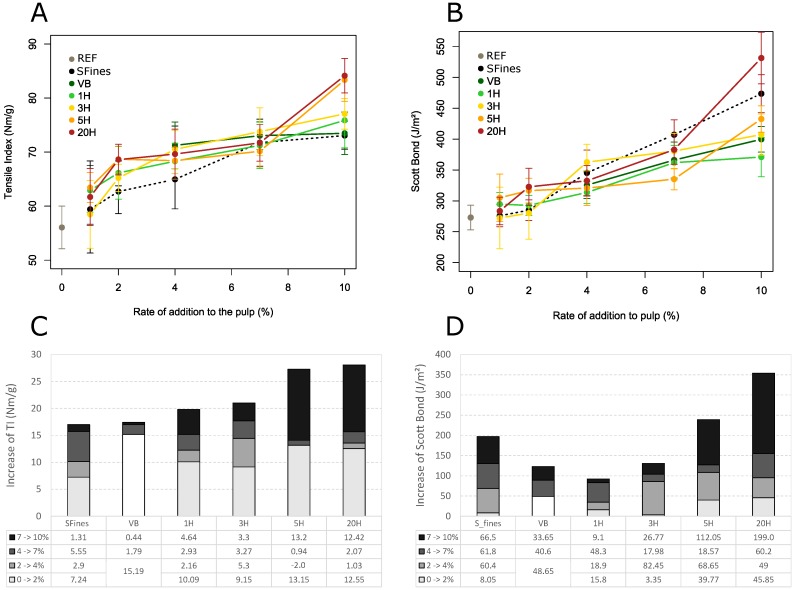
(**A**) Influence of the nature and quantity of the different fibrillated materials on tensile index (TI); (**B**) Influence of the nature and quantity of the different fibrillated materials on paper z-strength (Scott Bond); (**C**) Contribution of the partial increases of TI for each successive rate of addition (**D**) Contribution of the partial increases of Scott Bond for each successive rate of addition.

The structural modifications also have an impact on paper mechanical properties. Figure 8A illustrates the results obtained for tensile index (TI) for the different sheets made from the different blends. The results presented here illustrate the effect of each additive on the improvement of the paper properties, compared to the reference paper made from the refined pulp. The detailed table of the paper properties measured for each formulation (average data and standard deviation) is presented in the Supplementary Material (Table S1).

For tensile properties, the development of TI at increasing addition rate of the fine materials is divided into three parts:

First, up to 2% of addition, bringing fine fibrillated material to the pulp is highly efficient: it results in a significant increase of TI, regardless of the nature of the additive (Figure 8A). On Figure 8B, which displays the contribution of the partial increases of TI for each successive rate of addition, we can observe that a high proportion of the final TI increase is already achieved at 2%. Indeed, at 2% of addition, we can observe an increase of +10.43 Nm/g on average over all the materials, which is almost half of the total average TI increase for all materials. From similar observations, Zimmerman [28] defined the concept of “filling threshold”, concentration of additives corresponding to the formation of a network structure. At this specific point, most of the mechanical improvement is achieved. Studying the effect of the addition of MFC produced through homogenization with never dried softwood pulp, Sehaqui [25] also show that most of the mechanical improvement of the composites paper occur between 0 and 2% of addition. Later, Alcala [29] established this filling threshold for 2.25%, for nanofibrillated cellulose produced from bleached eucalyptus pulp through tempo-oxidation process. These results are really close to our observations for samples 1H to 20H and support the existence of filling threshold of paper structure of around 2% for cellulosic microfibrillated additives. On the contrary, the addition of SFines linearly increase from 0% to 7%, a result already reported by several authors [11,13,30,31]. In that case, this filling threshold is less visible and might reveal that the addition of SFines doesn’t enable the creation of new network but only consolidate the existent fiber-fiber bonds.

Second, between 2% and 7% addition rate, there is little effect on paper tensile properties. The increase of TI is less marked for almost all additives (see Figure 8C). This observation follows the trend already observed for air permeability measurements, where the effect of adding more additive was more pronounced at low addition rates and then decreases more slowly until reaching a certain level (Figure 7A). Here we can assume that passing the filling threshold of paper mentioned earlier, most of the structuring effect of the fibrillated materials to favor fiber-fiber bonding already occurred, meaning that only a small quantity of fibrillated material seems sufficient to reach a significant improvement of paper’s density and to noticeably consolidate its structure. Sehaqui [25] also observed that further NFC addition above 2% did not markedly increase paper strength, interpreted here as a poor improvement of inter-fibers stress transfer mechanisms in the elastic region.

The third and final phase of the development of TI shows an interesting effect, which is in contrast to the results of air permeability measurements. Between a 7% and a 10% addition rate, a further significant increase of tensile properties is observable, which strongly depends on the nature of the additives. For SFines and VB, characterized by the lowest fibril area, there is almost no change in TI from 7% to 10% of addition. For 1H and 3H, TI increases almost linearly between 4% and 10% addition rate. Alcala [29] mixed dried bleached eucalyptus pulp operated from 300 to 600 bar pressure at 60 °C with a high-pressure homogenizer (similar to the 3H additive produced in this study) with PVA matrix, in order to characterize the fibrillated additives intrinsic properties. The authors report a linear increase of tensile strength from 52 to 100 MPa from 0 to 4.5% of addition, which is coherent to the results we obtained (data not shown).

In contrast, the two materials 5H and 20H with the highest degree of fibrillation show a further significant increase of TI, which is similar to the increase obtained at low addition rates (average increase of TI between 7% and 10% of +13.2 and +12.42 Nm/g for 5H and 20H respectively). Thus, only at these high rates of addition, a clear dependency on the character of the fibrillated fine materials becomes evident: The higher the fibril area, the stronger the increase of TI. Comparing fines and MFC like additives, Manninen [30] also report an increase of TI at higher content only for MFC like additives but not for fines. Once again, this result tends to discriminate two different mechanisms between MFC and fines reinforcement ability.

**Figure 9:**
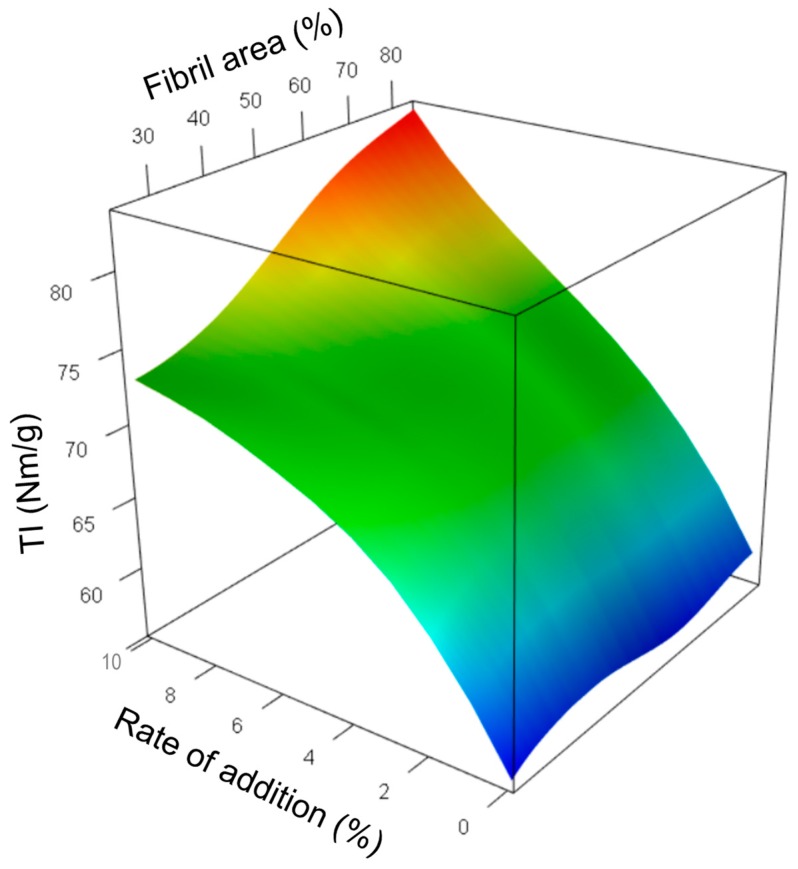
Combined influence of the morphological characteristics of the added materials and rate of addition on paper mechanical properties. Effect of fibril area and rate of addition on tensile index (TI).

Figure 9 illustrates the combined effect of both nature and quantity of the additive on paper tensile properties. Again, one can observe that the degree of fibrillation of the various material (represented here by the fibril area calculated from the microscopic images) has a more significant influence at high rates of addition. From 1% to 7%, TI shows a limited growth function with the rate of addition, which doesn’t depend on the fibril area. From 7% to 10%, a clear effect of quality of the additive is evident, with an almost linear increase of TI with Fibril Area. 

To explain this observation, we propose that two different phenomena come into play here:

First, the introduction of a low amount of fibrillated materials results in a noticeable improvement of the interactions between fibers. Paper strength is largely dependent on the number of fiber-fiber bonds formed during the consolidation and drying phases of the fiber network (handsheets were wet pressed at 150 bars during 90 s before drying in climate room). The tensile properties are therefore instantaneously improved. This hypothesis is supported by the work of several authors, who reported similar observations and explained the phenomenon with the following mechanism: the higher hydrogen bonding between the now larger bonded areas in molecular contact result in the increase of the cohesion between fibers in contact and therefore improve tensile strength. Sehaqui [25] for example, showed that the initial increase of TI up to 2% of MFC addition to the pulp also correspond to the largest decrease of paper porosity, supporting then the hypothesis of a “closing” of the structure which is achieved at 2% of addition of MFC. The author proposes at this step that the wood fibers are entirely coated of micro-fibrillated cellulose, as in the case of sisal reported by Juntaro [32].

**Figure 10:**
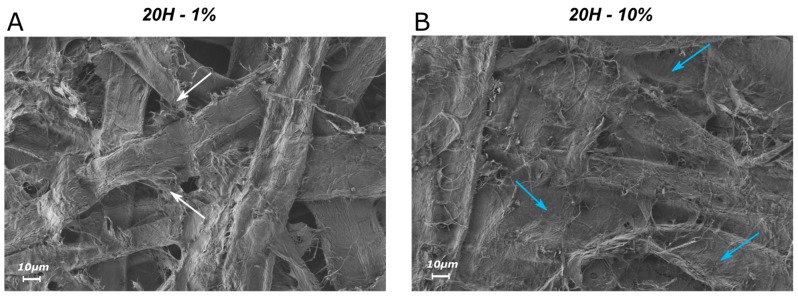
SEM images of the topography of composite handsheets realized with 1% (**A**) and 10% (**B**) of 20H (magnification 500×, with an acceleration voltage of 0.65 kV). The white arrows indicate zones where small fibrils, being likely the additives, act as connections between two fibers. The blue arrows indicate the areas forming smooth layer of fibrillar elements and entirely composed of additives, supposed to generate a secondary network in the material.

The SEM images of the paper samples also illustrate this mechanism (Figure 10A). In this image, showing the handsheets made with an addition of 1% of 20H, it is possible to identify several zones where small fibrils, being likely the added fibrillated material, act as connections between two fibers (indicated in Figure 10A by the white arrows). From the stabilization stage observed after 2%, we can thus infer that fibers are already well bonded to the adjacent ones at low addition rates. After that point, adding more fibrillated material to the fibers does not seem to be able to increase the bonded area to the same extent any more.

Secondly, at higher addition rates, the added fibrillated materials even further improve paper tensile strength depending on their degree of fibrillation. Here, the fibres’ surfaces might be entirely coated and saturated of fibrillar material. Bringing in a higher quantity of fibrillated material should not result in a further increase of the bonding area between the fibers at that point. We propose that passing 7% of addition, a second network of highly fibrillated material could be generated, resulting from an increase of bonding between the fibrillar elements. This hypothesis is supported by Sehaqui [25] who observed that strength and strain- to-failure is dramatically increased for the composition with 10% added NFC. At this point, additives might introduce new stress transfer mechanisms and energy-absorbing mechanism to failure events at a smaller length scale. In parallel to the filling threshold likely to occur at 2% of addition, 7% of addition seems to be another threshold corresponding to the structuration of the secondary network brought by the additive.

This hypothesis is supported by the SEM image of the surface of the composite paper made out of 10% of 20H additive (Figure 10B). Indeed, in this picture one can observe that the fibers are entirely covered by a smooth layer of fibrillar elements, with some area being entirely composed of additives (indicated in Figure 10B by the blue arrows). When the morphology of the added fibrous material is adapted by a higher degree of fibrillation to enable the establishment of second structural frame to the paper structure, this new parallel network is likely to bring higher tensile strength to the paper. The works of different other authors also observed similar changing in paper topography on SEM or AFM pictures when adding microfibrillar additives [4,23]. Taipale [23] proved that highly fibrillated additives show a more homogeneous structure and better interact with the fibers. Hii [4] proposed that higher amount of highly fibrillated, swollen and flexible MFC in the composites papers presumptively fills more paper porosity. Boufi [7] also proposed that MFC may generate a separate network embedded among larger fibers that contributes to boost the load-bearing capacity of the paper. Sehaqui [25], from SEM images with 10% of MFC proposed that MFC additives act as porous membranes or foams in the pores of the larger scale wood fiber network and conclude that “it becomes clear that dense regions of MFC contribute to substantial load-carrying ability to the material”. The different works cited above are important elements supporting our hypothesis. Finally, the fact that a linear correlation between fibril area and the increase of TI is observed at high addition rates leads to the conclusion that this second internal network generated by the added materials only leads to an improvement in tensile properties when the material is highly fibrillated. The fines fractions observed in SFines and VB, which showed the lowest degree of fibrillation, might not be able to interact and do not generate a strong cohesive network. On the contrary, the highly fibrillated fractions of 5H and 20H are likely to interact strongly, which could explain the significant increase of TI at 10% addition rate of these materials. Su [27] observed on SEM images that MFC additives at 10% of addition lead to a homogeneous covering of the fibers, which differ from the fibrillar aspect of composites papers made with 10% of refined fibers. We also report here that, contrary to highly fibrillar additives, SEM observations of composites papers made with 10% SFines and VB did not show similar smooth surface layers. In that case an irregular surface resulting from fibers-fibers bonding network was still visible.

Comparing nano and micro-scale additives obtained from softwood pulp through grinding or beating processes, Afra [33] observed that beating create a partial skin fibrillation while grinding turned fiber from micro to nanoscale through nanofibrillation mechanism and lead to more pronounced changes of paper properties when mixed to the pulp. The authors suppose that this result can be attributed to the reduction of defect points of cellulose fibers which increase the homogeneity of the structure, and to the higher ability of individual fibrils to attach and entangle cellulosic fibers, leading to a homogeneous and well-connected network.

Correlating this result to the work of Kang [34] who observed that the degree of internal fibrillation is linearly correlated with tensile index, while tensile index increases strongly with external fibrillation until a maximum and then stabilized, it is reasonable to propose that intensive homogenization processing leads to external fibrillation responsible for the homogeneous and strong fibrillar network observed at high addition rate. 

Additionally, MFC additives might also reduce stress concentration in the bonded region leading to more uniform stress distribution. Nanko [35] have specified two structural features of bonds where fibrillar additives can contribute to increased bond strength: the “bonding layer” between two fiber surfaces consisting of randomly oriented fibrils of fines material that fill the potential gaps between fibers at the bonded areas; and the “covering layer” at the periphery of a fiber bond protecting and consolidating the bonding edge susceptible to crack. Here the high homogeneity of these two features with highly mechanical treated additives might enable a nice continuity of these areas leading to a parallel fibrillar network. The fibril network might then improve load transfer between wood fibers as damage starts to develop, which could, as proposed also by Sehaqui [25], delay the development of large-scale damage sites to higher stress and strain. Finally, the effect of drying on paper strength is also to be considered. Jentzen [36] and later Lobben [37] and Wuu [38] identify that drying under tension induced by highly swollen material like MFC will also result in improved tensile strength. In our case we observed (data not shown) a good correlation between fibrillation rate and water retention of the materials which could lead to strong drying tensions during sheet formation.

All these elements might explain the difference observed depending on the quantity and quality of each additive studied in this work. The discussion of these results gives new keys for a better understanding of the intrinsic effect of each class of additives on paper strength and opens new perspectives in papermaking. Indeed, the concept of a new generation of papers with fibrous networks at two different scale levels is of interest for the development of new types of paper composites with improved mechanical performance.

## 4. Conclusion

We compared the effect of different fibrillated cellulosic additives produced from the same bleached softwood kraft pulp on paper properties, i.e., secondary cellulosic fines isolated from the pulp by means of fractionation in a laboratory pressure screen and MFC-like materials of increasing fibrillar character obtained by refining and high-pressure homogenization.

The microscopic techniques used in this work allowed the characterization of the morphological properties of the produced materials and allowed a better understanding of their structuring effect when used as additives in a paper furnish. The results showed that the fibril area as well as the size of the fractions (ECD) are key elements to be considered, but that their influence on paper properties varies depending on the quantity of additive added to the furnish. At low addition rates all of the applied MFC-like fine fibrous materials have a significantly positive effect on permeability and mechanical properties, which is more or less independent from the morphology of the materials. The closing of the paper structure observed here is of interest for packaging applications: enhanced barrier properties can be reached with a small quantity of additives. 

At low addition rates of fine cellulosic materials, it is not necessary to use highly fibrillated additives, which are costlier to produce, since highly fibrillated materials seem to only generate a slight improvement of paper properties. At higher addition rates, however, the additional effort to produce highly fibrillated and small material is worthwhile since a really significant improvement of final paper properties can be achieved.

These results also allow a better understanding of the involved mechanisms. Up to an addition rate of around 7% fine fibrous materials improve bonding between the areas in molecular contact more or less independently of their morphology and increase the cohesion between fibers in contact and therefore improve strength properties. Up to this addition rate the key parameter is the rate of addition, which correlates to the improvement of all paper properties determined in this study. At higher addition rates up to 10% the morphological character of the added fine fibrous materials strongly influences the results. A significant positive effect of fibril area on tensile strength becomes evident. These observations could be explained by the creation of a second network structure of highly fibrillated materials at high addition rates, which improves the load-bearing capacity of the whole paper structure, leading to higher tensile strength. 

In order to verify these hypotheses, further studies are planned to identify the exact location of the fine cellulosic materials in the paper network depending on their morphology. Also, the intrinsic mechanical properties of the proposed secondary network built up by fine cellulosic materials with different degrees of fibrillation and size will be evaluated by determining the mechanical properties of handsheets of different basis weights. Finally, in the view to complete this work, it would also be interesting to study the effect of MFC like additives on wet strength, which might be improved thanks of the gel-like behavior of swollen highly fibrillated materials like the one we produced.

## Figures and Tables

**Table 1:** Detail of the materials used in the study with corresponding treatments.

Material	Sample	Fractionation	Beating	Homogenization
Reference SBK Pulp	REF	-	PFI mil to 16SR	-
Secondary Fines	SFines	Pressure screen 100 µm	Refining in an industrial disc refiner to 38SR	-
Valley Beater pulp	VB	-	2 h of Valley Beater to ~90° SR	-
VB+1 Step of homogenization	1H	-	2 h of Valley Beater	300 bars
VB+3 Steps of homogenization	3H	-	2 h of Valley Beater	300, 400, 500 bars
VB+5 Steps of homogenization	5H	-	2 h of Valley Beater	300, 400, 500, 600, 700 bars
VB+20 Steps of homogenization	20H	-	2 h of Valley Beater	300 to 600 bars and 16 times 700 bar

**Table 2:** Average values of Equivalent Circular Diameter (ECD) and Length (weight weighted) obtained for refined pulp and each fine cellulosic materials. The values were calculated from the distributions of the three measurements realized per material, using L&W Fiber Tester+.

Material	ECD [µm]	Length [µm]
Reference refined pulp	244.1	2478.3
SFines	50.6	185.3
VB	141.5	1226.9
1H	122.5	1073.9
3H	100.8	873.7
5H	75.3	607.9
20H	53.6	379.5

## Supplementary Materials

The following are available online at https://www.mdpi.com/2079-4991/9/3/321/s1, Table S1: Table of the averaged properties measured for each composite paper.
