# Supplementary material for: Fine Cellulosic Materials Produced from Chemical Pulp: the Combined Effect of Morphology and Rate of Addition on Paper Properties
Source: Nanomaterials (Basel). 2019 Mar 1;9(3):321. doi: 10.3390/nano9030321 (PMC6473348; doi:10.3390/nano9030321)
Supplement: Supplementary file 1 [file nanomaterials-09-00321-s001.pdf]

Supplementary Materials

Table of the averaged properties measured for each composite paper.

		Thickness [μm]		Basis Weight [g/m²]		Density [g.cm⁻³]		Air permeability [ml/min]		Tensile Index [Nm/g]		Scott Bond [J/m²]	
**Materials**	**Addition [%]**	**Average**	***St. Dev.***	**Average**	***St. Dev.***	**Average**	*St. Dev.*	**Average**	*St. Dev.*	**Average**	*St. Dev.*	**Average**	*St. Dev.*
**Reference refined pulp**	-	**99.0**	*2.46*	**59.6**	*0.51*	**0.60**	*0.21*	**2283.0**	*155.80*	**56.1**	*3.95*	**276.9**	*22.99*
**Valley Beater**	4	**91.9**	*4.75*	**60.2**	*0.99*	**0.65**	*0.21*	**577.0**	*29.00*	**71.3**	*4.32*	**325.5**	*21.69*
	7	**91.7**	*5.93*	**60.2**	*0.65*	**0.66**	*0.11*	**273.0**	*20.00*	**73.0**	*2.56*	**366.1**	*29.17*
	10	**91.4**	*4.57*	**61.1**	*0.60*	**0.67**	*0.13*	**126.0**	*4.00*	**73.5**	*3.93*	**399.8**	*20.80*
**Secondary Fines**	1	**100.0**	*1.74*	**60.3**	*1.09*	**0.60**	*0.63*	**1737.5**	*67.30*	**59.4**	*6.90*	**275.8**	*17.76*
	2	**100.5**	*2.66*	**60.7**	*0.62*	**0.60**	*0.23*	**1423.8**	*40.70*	**63.3**	*4.90*	**284.9**	*16.65*
	4	**97.4**	*2.14*	**60.9**	*0.83*	**0.63**	*0.39*	**737.0**	*60.40*	**66.2**	*5.00*	**345.3**	*36.86*
	7	**94.5**	*2.08*	**59.8**	*1.00*	**0.63**	*0.48*	**374.1**	*20.20*	**71.8**	*4.35*	**407.2**	*24.14*
	10	**94.2**	*2.12*	**60.9**	*0.88*	**0.65**	*0.42*	**162.0**	*18.30*	**73.1**	*2.52*	**473.7**	*30.69*
**1H**	1	**99.0**	*1.81*	**60.5**	*1.24*	**0.61**	*0.69*	**1462.0**	*93.40*	**62.9**	*4.05*	**294.7**	*18.96*
	2	**97.4**	*3.01*	**60.4**	*2.76*	**0.62**	*0.92*	**903.7**	*55.60*	**66.2**	*4.87*	**292.6**	*16.21*
	4	**96.7**	*1.78*	**59.6**	*0.57*	**0.62**	*0.32*	**483.0**	*19.70*	**68.3**	*3.36*	**313.6**	*17.93*
	7	**98.9**	*2.38*	**61.9**	*0.54*	**0.63**	*0.23*	**162.0**	*8.30*	**71.2**	*4.27*	**361.9**	*27.88*
	10	**96.7**	*2.87*	**60.3**	*0.68*	**0.62**	*0.24*	**89.3**	*3.10*	**75.9**	*5.08*	**371.0**	*31.77*
**3H**	1	**100.3**	*1.80*	**61.2**	*0.40*	**0.61**	*0.22*	**1767.7**	*78.20*	**58.5**	*6.27*	**272.2**	*49.84*
	2	**97.2**	*1.70*	**59.2**	*1.10*	**0.61**	*0.65*	**1151.5**	*43.20*	**65.2**	*2.53*	**280.2**	*42.20*
	4	**95.1**	*1.90*	**60.2**	*0.80*	**0.63**	*0.42*	**412.7**	*35.50*	**70.5**	*3.53*	**362.7**	*29.01*
	7	**94.5**	*2.40*	**60.0**	*0.90*	**0.63**	*0.38*	**157.7**	*7.60*	**73.8**	*4.42*	**380.6**	*28.80*
	10	**95.6**	*2.70*	**60.7**	*0.70*	**0.63**	*0.26*	**82.4**	*3.60*	**77.1**	*2.84*	**407.4**	*33.30*
**5H**	1	**99.4**	*1.70*	**59.3**	*0.40*	**0.60**	*0.24*	**1844.8**	*99.80*	**63.4**	*2.78*	**305.1**	*21.79*
	2	**97.7**	*2.00*	**59.8**	*0.60*	**0.61**	*0.30*	**1250.4**	*68.90*	**69.2**	*3.70*	**316.6**	*20.11*
	4	**98.0**	*1.90*	**60.0**	*0.60*	**0.61**	*0.32*	**457.7**	*37.70*	**67.2**	*3.64*	**320.7**	*28.12*
	7	**93.3**	*1.60*	**50.1**	*0.30*	**0.60**	*0.19*	**253.5**	*21.10*	**70.2**	*2.92*	**335.2**	*17.21*
	10	**92.0**	*1.80*	**60.1**	*0.40*	**0.65**	*0.22*	**47.3**	*4.10*	**83.4**	*3.97*	**432.7**	*21.40*
**20H**	1	**98.0**	*2.20*	**59.7**	*0.50*	**0.61**	*0.23*	**1311.0**	*120.40*	**61.7**	*5.27*	**283.5**	*22.36*
	2	**97.8**	*1.90*	**60.2**	*0.40*	**0.62**	*0.21*	**769.2**	*79.40*	**68.6**	*2.85*	**322.7**	*30.06*
	4	**96.3**	*2.90*	**59.6**	*0.50*	**0.62**	*0.17*	**303.0**	*50.80*	**69.6**	*4.61*	**332.5**	*24.75*
	7	**93.8**	*1.70*	**59.7**	*0.50*	**0.64**	*0.29*	**108.2**	*14.70*	**71.7**	*3.38*	**382.9**	*23.59*
	10	**87.4**	*1.70*	**61.5**	*0.60*	**0.70**	*0.35*	**30.4**	*4.00*	**84.1**	*3.18*	**531.5**	*41.91*
